# The Role of Food in the Treatment of Bowel Disorders: Focus on Irritable Bowel Syndrome and Functional Constipation

**DOI:** 10.14309/ajg.0000000000001767

**Published:** 2022-04-08

**Authors:** Prashant Singh, Caroline Tuck, Peter R. Gibson, William D. Chey

**Affiliations:** 1 Division of Gastroenterology and Hepatology, Department of Internal Medicine, University of Michigan, Ann Arbor, Michigan;; 2 Department of Dietetics, Nutrition and Sport, La Trobe University, Bundoora, Australia;; 3 Department of Gastroenterology, Central Clinical School, Monash University, Melbourne Victoria, Australia.

## Abstract

Irritable bowel syndrome (IBS) and functional constipation (FC) are among the most common disorders of gut–brain interaction, affecting millions of individuals worldwide. Most patients with disorders of gut–brain interaction perceive food as a trigger for their gastrointestinal symptoms, and specific dietary manipulations/advice have now been recognized as a cornerstone therapeutic option for IBS and FC. We discuss in detail the 2 most common dietary interventions used for the management of IBS-general dietary advice based on the National Institute for Health and Care Excellence guidelines and a diet low in fermentable oligosaccharides, disaccharides, monosaccharides, and polyols (FODMAPs). We summarize the literature around the possible mechanisms of FODMAP-mediated IBS pathophysiology, the current 3-step, top-down approach of administering a low FODMAP diet (LFD) (restriction phase, followed by reintroduction and personalization), the efficacy data of its restriction and personalization phases, and possible biomarkers for response to an LFD. We also summarize the limitations and challenges of an LFD along with the alternative approach to administering an LFD (e.g., bottom-up). Finally, we discuss the available efficacy data for fiber, other dietary interventions (e.g., Mediterranean diet, gluten-free diet, and holistic dietary interventions), and functional foods (e.g., kiwifruit, rhubarb, aloe, and prunes) in the management of IBS and FC.

## INTRODUCTION

The Rome IV process identified 5 separate but overlapping bowel disorders, including irritable bowel syndrome (IBS), functional constipation (FC), functional diarrhea, functional bloating/distension, and unspecified functional bowel disorder (1). Of these conditions, FC and IBS are 2 of the most prevalent, affecting 11.7% and 4.1% in a recent survey of more than fifty-four thousand individuals from all over the world (2). Patients with bowel disorders often identify food as an important trigger for their gastrointestinal (GI) symptoms. For example, in a survey of nearly 200 patients with IBS from Sweden, 84% identified food as a key trigger for their GI symptoms (3). The reasons that underlie the relationship between food and the development of GI symptoms are discussed in detail in another manuscript (4). In many patients with meal-related GI symptoms, diet manipulation is a natural first step in the treatment plan. At present, the greatest proportion of the literature addressing diet interventions to treat bowel disorders focuses on IBS and FC. In this article, we summarize the evidence that supports usual dietary advice, fiber supplementation, a diet low in fermentable oligosaccharides, disaccharides, monosaccharides and polyols (FODMAPs) (a low FODMAP diet [LFD]), other emerging holistic and targeted dietary interventions, and functional foods for these conditions.

## DIETARY INTERVENTIONS FOR IBS

### General dietary advice

First-line dietary management strategies for patients with IBS and other bowel disorders include healthy eating habits such as those outlined by the National Institute for Health and Care Excellence (NICE), United Kingdom (5), with similar recommendations made by the British Dietetic Association (6) (Table 1). Both sets of guidelines are based on low and moderate quality evidence, and, despite widespread use and acceptance of these recommendations, there have been no randomized controlled trials (RCTs) comparing this approach with habitual or sham dietary interventions.

**Table 1.**
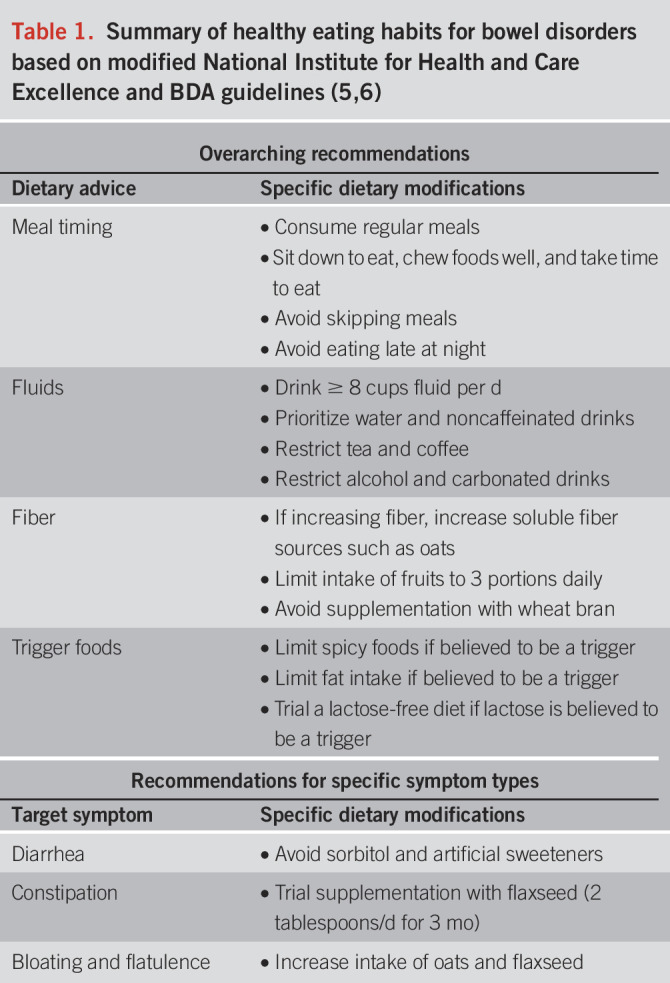
Summary of healthy eating habits for bowel disorders based on modified National Institute for Health and Care Excellence and BDA guidelines (5,6)

Both NICE and British Deitetic Association (BDA) guidelines recommend these healthy eating strategies as a first-line therapy for patients with IBS, with the low FODMAP diet reserved for those with persistent symptoms. However, a recent meta-analysis found that the NICE guidelines were not superior to any of the alternative or control dietary interventions analyzed (7). This was in contrast to individual trial results whereby the NICE guidelines had similar efficacy to LFD, providing adequate relief in 41% of IBS with diarrhea (IBS-D) participants in the United States (8) and reducing total IBS-severity scoring system (IBS-SSS) in 46% of participants with IBS in Sweden (9). Despite this, other considerations, such as ease of implementation and more broad health benefits of the NICE guidelines, suggest they are still of importance as a first-line therapy in managing bowel disorders.

### Fiber supplementation

Dietary fiber comprises a diverse group of nondigestible carbohydrates containing varying length chains of sugar monomers. Fiber is characterized by heterogeneity in structure, functional properties including bulking, viscosity/gel formation, and fermentability (Table 2), and clinical effects (10). Dietary fiber represents a wide variety of fiber types with varied functional properties (11). Despite how commonly fiber is the target of manipulation in clinical practice, the effect of systematically altering dietary fiber intake in patients with IBS has not been formally reported. The NICE guidelines recommend limited intake of high-fiber foods (e.g., whole meal bread) and resistant starch (e.g., processed or recooked foods), largely on the basis of expert opinion. Most clinical evidence relates to the use of specific fiber types as supplements, where monotherapy with psyllium (ispaghula) or wheat bran have been the main fibers assessed in RCTs. Meta-analyses have reported symptomatic benefit only for psyllium (7–30 g/d, number needed to treat (NNT) = 7) and not for wheat bran (12), inulin, or oligosaccharides (13). Based on these findings, clinical practice guidelines have recommended the use of soluble fibers and avoidance of insoluble fibers for patients with bowel disorders such as IBS and chronic idiopathic constipation. Unfortunately, dichotomizing the benefit of fiber on the basis of solubility oversimplifies the many ways in which fiber can influence the luminal microenvironment and gut function (10,14).

**Table 2.**
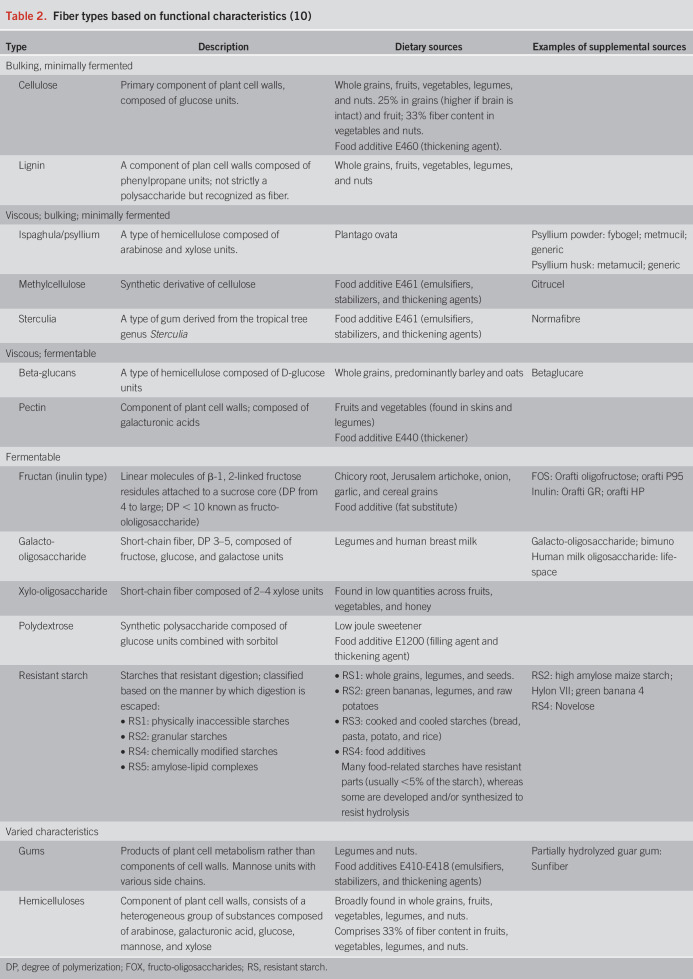
Fiber types based on functional characteristics (10)

The goals of introducing fiber supplements to patients with IBS are 4-fold. First, fibers have been applied to normalize stool characteristics. For example, fibers with particulate and water retention properties (such as wheat bran or sugarcane bagasse (10)) may hasten colonic transit time and increase fecal bulk in patients with slow colonic transit, whereas fibers with viscous characteristics (such as psyllium) have been better for normalizing stool form. Second, fibers, through direct and indirect effects, may improve the structure and function of the gut microbiota. As substrates for fermentation, dietary fibers may be associated with benefits to gut health from, for example, delivery of short-chain fatty acids to the colonic mucosa (15). Currently, such suggestions are largely aspirational, given the lack of supportive outcomes data. Third, fibers can be used to correct or prevent problems associated with other diet therapies, especially the LFD, which tends to reduce fiber intake, potentially leading to suboptimal benefits for stool characteristics and reduced fermentation in the distal colon (16). Finally, a major goal when initiating fiber supplementation is to avoid exacerbating IBS symptoms, which presents as a real risk for fibers that contain readily fermentable and, hence, gas-producing components, such as fructans alone or when present in wheatbran and resistant starch. The use of nonfermented or very slowly fermented fibers such as sugarcane bagasse and psyllium are relatively well tolerated (17,18) and both, by virtue of slowing fermentation and the rate of gas production, may be well tolerated when used with fermentable fiber (17–19). Clinical experience indicates, however, that a gradual introduction of additional fiber is better tolerated than initiating a large dose.

## THE LFD

### Pathophysiology

**Figure 1.**
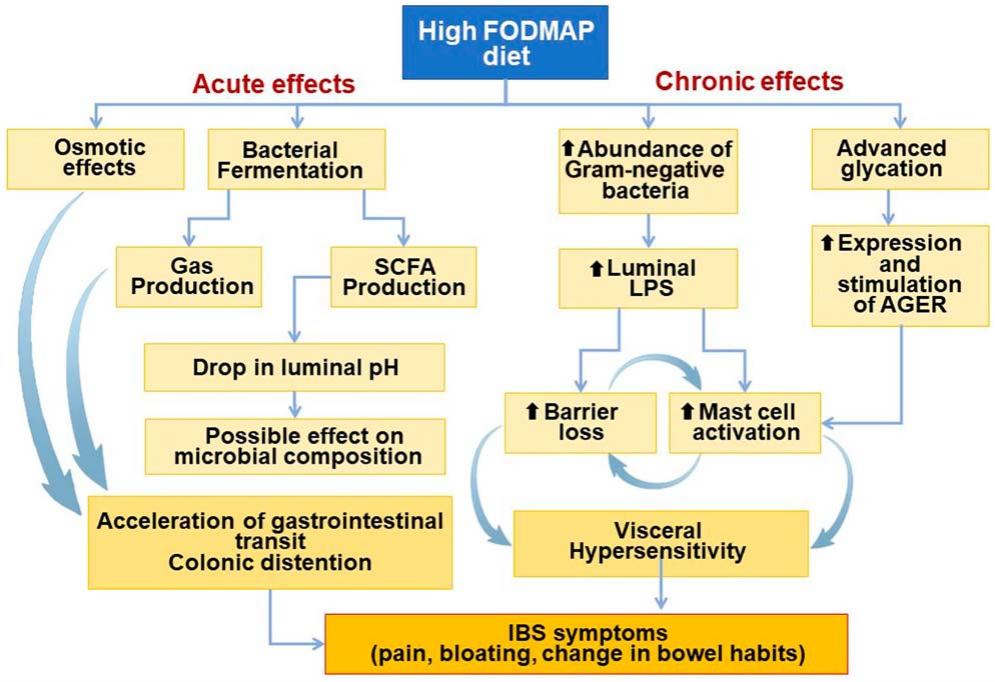
FODMAPs exert multiple effects in the GI tract.

Initially, the effects of FODMAPs on gut physiology were believed to be primarily due to stimulation of mechanoreceptors as a response to luminal distention (20). While fructose distends the small bowel with water due to its osmotic effects, fructans distends the colon from release of gases (such as hydrogen and methane) due to bacterial fermentation (21). However, recent studies indicate that their contribution to IBS pathophysiology is much more complex (Figure 1). Rodent studies suggest that a high FODMAP diet can cause dysbiosis, colonic barrier dysfunction, recruitment and activation of mast cells, and visceral hypersensitivity (22–24). Two pathways of FODMAP-mediated visceral hypersensitivity have been proposed. In rodents, a high FODMAP diet leads to an abundance of Gram-negative bacteria that increase luminal lipopolysaccharide (LPS). LPS can activate mast cells through toll-like receptor 4 to release bioactive molecules such as tryptase, histamine, and prostaglandin E2,^23^ which can increase intestinal permeability and visceral sensitivity (12,23). Mouse studies have also reported that FODMAP-mediated visceral hypersensitivity is associated with an increased expression of advanced glycosylation end product–specific receptor and is ameliorated in the presence of an antiglycation agent (24).

*In vitro* studies using a mouse model indicate that fecal supernatants from patients with IBS-D on a high FODMAP diet significantly increase mast cell activation compared with fecal supernatants from healthy controls (23). This effect is ameliorated in the absence of toll-like receptor 4 and after an LFD (23). Conversely, 2 small studies in patients with IBS-D found that an LFD led to significantly reduced fecal LPS levels (22,23) and an increased colonic expression of tight junction proteins and decreased markers of mast cell activation, including serum histamine and tryptase (22,23). While these observations expand the range of possible reasons that FODMAPs might cause GI symptoms, they require further clinical validation before concluding that there is a clear cause and effect relationship.

### Efficacy data

In the seminal feeding trial, Halmos et al. (22) performed a single-blind, crossover RCT in which 30 patients with IBS were randomized to an LFD vs a typical Australian diet for 21 days. The primary endpoint was overall GI symptoms measured using a 0–100 mm visual analog scale. The study found that the overall GI symptom score was significantly lower in the LFD group compared with that found in the typical Australian diet group (22.8 vs 44.9, *P* < 0.001) (25). Subsequent to this trial, numerous RCTs have investigated the efficacy of LFD in patients with IBS. A recent network meta-analysis pooled data from 13 RCTs evaluating the efficacy of LFD in IBS and found that LFD was superior to other dietary interventions in achieving improvement in global IBS symptoms, abdominal pain, and bloating (7). However, this network meta-analysis did not find LFD superior to other dietary interventions in achieving an improvement in bowel habits in IBS even if the analysis was restricted to patients with IBS-D (7).

### Low-FODMAP diet compared with other active diet interventions

Given the difficulty in blinding and using a true placebo in dietary intervention studies, several have compared LFD with another active dietary intervention. A multicenter, parallel group, single-blind RCT from Sweden compared a dietitian-led LFD with standard dietary advice (based on the NICE guidelines) over 4 weeks in 75 patients with IBS (9). Both groups experienced significant improvement in symptom severity (measured using IBS-SSS) at the end of the 4 weeks compared with that in baseline (*P* < 0.001), without a difference between the groups (*P* = 0.62) (9). In a US study, 84 patients with IBS-D were randomized to an LFD or modified NICE (mNICE) diet for 4 weeks (8). Fifty-two percentage of the patients in the LFD group reported an adequate relief of overall IBS symptoms compared with 41% in the mNICE group; the difference between the groups was not statistically significant (*P* = 0.31). However, an LFD resulted in a significantly higher proportion of abdominal pain and bloating responders compared with those in the mNICE group (*P* < 0.01(8) for both comparisons). This study also reported improvements in IBS-related quality of life and reductions in activity impairment with LFD compared with those with the mNICE diet (26).

Three studies have compared the efficacy of an LFD with a traditional diet based on the NICE guidelines in regions not consuming a western diet (e.g., Iran, India, and China (27–29)). While 2 of these studies reported significantly greater improvement in GI symptoms with an LFD (27,28), 1study did not find a significant difference between the groups (29). Finally, the recent network meta-analysis discussed earlier found LFD superior to a diet based on the NICE guidelines for global IBS symptoms, abdominal pain, and bloating (7).Recently, smartphone app-based delivery of LFD was shown superior to medical therapy with otilonium bromide in a large RCT of 453 primary care patients with IBS. Seventy-one percentage of the patients in the LFD group responded (defined by a 50-point decrease in IBS-SSS) compared with 61% in the medical therapy group (*P* = 0.03) (30).

### Low FODMAP diet compared with placebo/sham diet

In a multicenter, randomized, placebo-controlled trial from the United Kingdom, a sham diet of similar complexity, intensity, and fiber/energy was compared with an LFD (31). Although the percentage of participants reporting adequate symptom relief in the intention-to-treat analysis did not reach statistical significance (57% in the LFD group vs 38% in the sham diet group, *P* = 0.051), the difference was significant in the per-protocol analysis (61% vs 39%, *P* = 0.042 (31)). In addition, the IBS-SSS for the LFD group was also significantly lower than that for the sham group (31). In a second multicenter, randomized, placebo-controlled trial from the United Kingdom, patients had 2.3-fold higher odds of achieving adequate symptom control with an LFD compared with that for a sham LFD, although this did not reach statistical significance (32).

### LFD vs high FODMAP diet

Only one study by McIntosh et al. (33) compared an LFD with a high FODMAP diet in 40 patients for 3 weeks and found a significant reduction in IBS symptom severity with an LFD, whereas a high FODMAP diet led to a mild increase in IBS symptom severity.

### The LFD is a 3 step program

**Figure 2.**
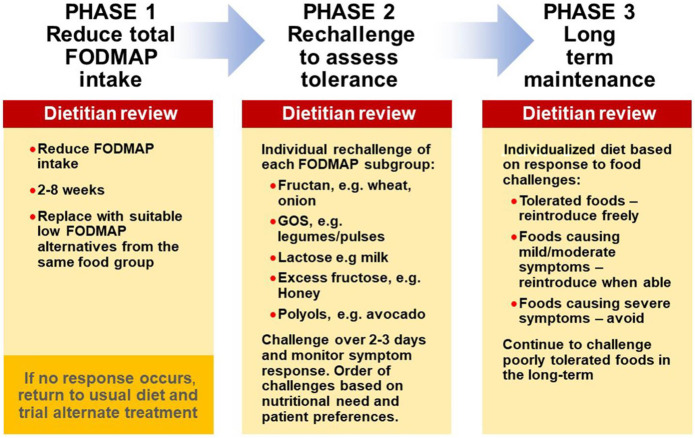
The low FODMAP diet is a three step process.

The LFD is a 3-step process involving an initial 2- to 6-week restriction phase (phase 1), followed by a rechallenge phase (phase 2) to identify food triggers, including dose tolerated, and, finally, a long-term maintenance (personalized) phase based on the outcome of the rechallenges (phase 3) (34) (Figure 2). Owing to the restrictive nature of phase 1, rechallenge to identify specific triggers in the individual and allow maximal reintroduction of tolerated foods is imperative to the long-term success of the diet. Phase 1 involves the reduction of high FODMAP foods such as wheat, onion, garlic, apples, and pears, with simultaneous replacement of suitable low FODMAP alternatives ideally from the same food group. Phase 1 should only be followed for as long as necessary to ascertain whether symptom response will occur (usually 4–6 weeks). Phase 2 enables patients to identify specific food triggers and reintroduce tolerated foods back into the diet (35). While FODMAP intake has been shown to increase during phase 2 (12 ± 8 g/d vs 22 ± 11 g/d, *P* < 0.01), symptom control is ideally sustained (36). Likewise, in phase 3, symptomatic improvement typically continues at 12 months (adequate relief achieved in 67% vs 28% at baseline, *P* = 0.04) (37). Of importance, when the patient has not had guidance from a dietitian, adherence with phases 2 and 3 has been shown to be poor (phase 2: 70% vs 39% compliant, *P* = 0.02; phase 3: 65% vs 29% compliant, *P* < 0.01), and as such, it is recommended the diet be guided by an adequately trained dietitian (38). That said, future studies may assess the use of new technologies, such as mobile applications, which may change the way the diet is delivered and patients are monitored, especially where access to dietitians is limited (39,40).

**Figure 3.**
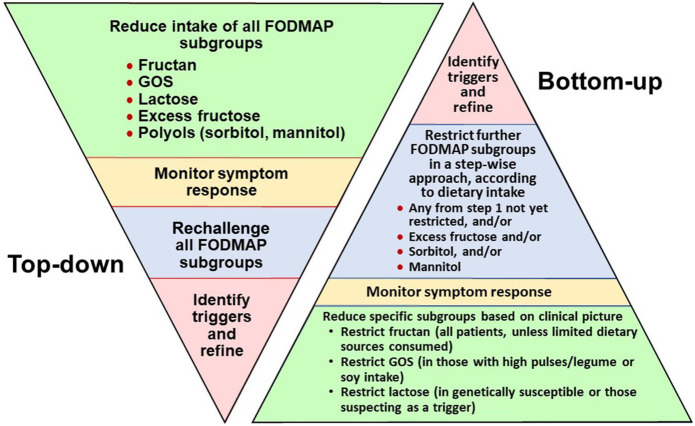
“Top-down” or “Bottom-up” approaches to the low FODMAP diet.

Each phase of the 3-step ‟top-down” LFD should be implemented in a personalized manner to maximize benefits and minimize restrictions. However, an alternative approach exists whereby only a few specific FODMAP subgroups are restricted based on diet history and ethnic risk profiles (41). This approach, termed bottom-up or FODMAP gentle, restricts only 1 or 2 FODMAP subgroups initially, evaluating symptom response and continuing to restrict further only if required (37). Emerging data suggest that fructans, mannitol, and galacto-oligosaccharides are reportedly the most consistent FODMAP subgroup to trigger symptoms (42,43), and lactose may be helpful to restrict in genetically susceptible individuals, although this remains controversial (41); hence, these may be most relevant to restrict initially (Figure 3). While only limited data exist for this approach, it may be best suited for those with milder symptoms, nutritional deficiencies, or at risk of disordered eating. The traditional top-down approach may be more challenging to undertake in the initial weeks, but following the rechallenge (phase 2) and maintenance (phase 3) phases, it may be better able to identify specific food triggers and hence improve the long-term success of the diet, although this has not been specifically studied.

### Long-term data

All the above-mentioned studies have investigated the efficacy of the restriction phase of an LFD. However, a few studies suggest that the restriction phase may be associated with reduced dietary intake of some micronutrients (e.g., iron and thiamine) and may lead to a reduced fecal abundance of putatively beneficial bacteria such as *Bifidobacteria* spp. (44–46). Given its restrictive and cumbersome nature, the restriction phase is not a long-term strategy and, in responders, should always be followed by the reintroduction and personalization phases.

Recent prospective studies have investigated the long-term effectiveness of a personalized LFD (37,47–49). These studies show that up to 80% of patients with IBS patients are on a personalized LFD 6–12 months after the restriction phase with 57%–67% of patients reporting adequate/satisfactory relief of IBS symptoms (37,47,48). A small study (n = 41) with a mean follow-up of >12 months reported that a personalized LFD led to significant improvement in the quality of life and anxiety scores (49). Although some studies have raised concerns about inconvenience, nutritional deficiencies (45,49) and incremental costs with the restriction phase of LFD, no difference in total energy intake, macronutrient, and micronutrient intake between individuals on a personalized LFD were compared with those on a habitual diet (47). In another long-term follow-up study of an RCT, a personalized LFD did not result in differences from baseline in the abundance of potentially beneficial bacteria such as Bifidobacteria (37).

Overall, studies indicate that most patients with IBS who respond to LFD will be able to liberalize their diet if they complete all 3 phases of the LFD program. Available studies suggest that benefits to overall IBS symptoms are durable over an extended follow-up and when dietitian led, have only minor effects on macronutrient/micronutrient intake.

### Emerging biomarkers to predict response

#### Volatile organic compounds.

In an RCT of LFD vs sham diet, baseline fecal volatile organic compound profiling contained 15 features that classified response to the low FODMAP diet with a mean accuracy of 97% (95% confidence interval (CI), 96%–99%) (50), although no validation study has been reported.

#### Microbiome markers.

In a 2-day double-blind, crossover, feeding trial in children with IBS, the fecal microbiome of responders was found to be enriched in bacteria known for saccharolytic metabolic capacity (51). Others have also reported a higher abundance of saccharolytic bacteria among LFD responders in adult patients with IBS. In a parallel-group, RCT of 4-week LFD vs NICE diet in 67 adult patients with IBS, nonresponders to LFD were found to have a higher dysbiosis index score at baseline compared with LFD responders (52). An open-label 4-week LFD intervention study reported that 10 of 54 bacterial markers included in a commercially available GA-map Dysbiosis test differed significantly between responders and nonresponders (53). Recently, a pathogenic microbial signature with a decrease in *Bacteroidetes* spp., an enrichment of *Firmicutes* spp., and genes fsor carbohydrate metabolism was identified in up to 50% of patients with IBS. Dietary FODMAP restriction tended to improve this dysbiosis and normalize the metabolic gene pathways (54). However, not all studies have found significant differences between the fecal microbial composition of LFD responders vs nonresponders (55).

### Other diet interventions

Several diets for patients with IBS are available, and most do not have compelling evidence of effectiveness. A summary of dietary strategies, evidence, and issues is listed in Table 3. In general, 2 approaches have been taken:

**Table 3.**
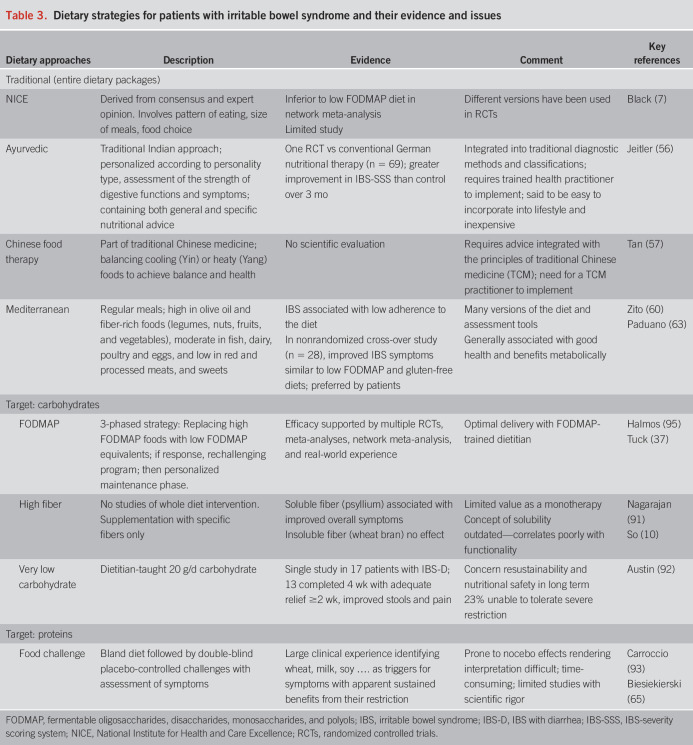
Dietary strategies for patients with irritable bowel syndrome and their evidence and issues

### Holistic dietary plans

These involve recommendations across many food groups and, for some, across eating habits and practices. The major difficulties in investigating such diet plans are that they are not standardized and findings in studies may not be generalizable. It should come as no surprise that controlled evidence for their benefit is generally lacking, although it must be conceded that a lack of evidence neither proves nor disproves benefit. Ancient health systems, foreign to modern medicine, such as Indian Ayurveda (56) and Traditional Chinese Medicine (57), implement dietary change to improve health status, including gut symptoms. Ayurvedic dietary approaches were subjected to a randomized, controlled comparison with conventional German nutritional therapy, itself uncertain in efficacy, and showed greater symptomatic improvement in patients with IBS (56).

The Mediterranean diet was never designed as a therapeutic diet for IBS, but it is believed to confer broad health benefits including reduced cardiometabolic risk and all-cause mortality (58). The diet encourages regular meals and is high in olive oil and fiber-rich foods, moderate in fish, dairy, poultry, and eggs, and low in red and processed meats and sweets (58,59). It may be beneficial in reducing bowel symptoms due to its positive impact on the gut microbiota and lower intake of saturated fat, proposed to reduce microscopic inflammation and regulation of the gastro-colonic reflex (59–61). The presence of IBS has been associated with low (odds ratio (OR) = 3.24, 85% CI: 1.73–6.08, *P* < 0.0001) and intermediate (OR = 1.91, 95% CI: 1.14–3.22, *P* < 0.05) adherences to a Mediterranean diet (60). Lower adherence has been associated with more severe abdominal pain and flatulence in patients with IBS (62). In 28 participants with IBS who trialed 4 weeks each of an LFD, followed by gluten-free and then balanced Mediterranean diet, all 3 diets improved global symptoms (*P* < 0.01), abdominal pain (*P* < 0.01), and bloating (*P* < 0.01) (63). While LFD provided superior reductions in bloating, the balanced Mediterranean diet had the highest levels of participant acceptance (63). However, significant methodologic limitations include a lack of randomization, blinding, or assessment of adherence. Therefore, data to date are insufficient to support routine use of the Mediterranean diet in bowel disorders, but the potential for benefit warrants further investigation.

### Diets targeting specific food types or components

The pathogenic involvement of low-grade gut inflammation with increased numbers and activation of intraepithelial lymphocytes, mast cells, and eosinophils has stimulated interest in gut-specific hypersensitivity responses to dietary proteins (64). Identification of such proteins could enable personalized dietary recommendations. Three targeting methodologies have been described to date.

Double-blind placebo-controlled challenges have been unpopular due to their resource intensity and problems with overestimation of cause and effect resulting from the nocebo response, as has been common in gluten challenges (65). Second, proteins with known pathogenic potential can be assessed in patients with IBS by withdrawal–rechallenge methodology. Such an approach has been assessed with gluten with the emergence of a new condition of nonceliac gluten or wheat sensitivity. Unfortunately, a gluten-free diet also reduces other potential triggers of gut symptoms, especially fructans, and response to such a dietary trial does not mean that gluten is the cause of symptoms. Indeed, a blinded cross-over rechallenge study in patients with IBS who responded to a gluten-free diet indicated fructans rather than gluten as the main culprit for inducing symptoms (66). A biomarker that identifies wheat-related proteins as causally related to symptoms is needed. Third, specific immune reactions to food antigen exposure might better identify problem foods. Gut-specific reactions have been demonstrated in patients with food-related symptoms by the demonstration of IgE and mast cell activation associated with intramucosal injection of food antigens (64) and by the direct observations of injury response using confocal laser endomicroscopy after topical application of specific food antigens to the duodenum and an intramucosal injection of antigens during colonoscopy (67–70). Both methods have provided evidence that dietary restriction of implicated antigens led to clinical benefits for patients. These 2 methods are expensive, invasive, and present technical challenges, but are leading us toward a better understanding of food–IBS relationships. The key question is whether such antigens can be identified in the systemic immune compartment. Standard allergy testing (e.g., skin tests, food-associated IgE, and basophil activation) are not useful but claims that levels of food-related IgG (71) and volumetric responses of leukocytes to the antigens *in vitro* (72) do identify proteins with pathogenic significance in the gut of patients with IBS. Although interesting, these techniques have not achieved wide acceptance for 2 very important reasons. First, the specificity of the findings to IBS and relationship to symptom genesis is not well substantiated, and second, peer-reviewed scientific evaluation of the effect of diets guided by the findings is scarce.

The other target for dietary manipulation is food-associated bioactive chemicals that are naturally occurring or introduced into the food supply. To date, such concepts have received limited scientific evaluation. A low chemical diet that uses an elimination–rechallenge approach is reported anecdotally to provide benefit but has not been subjected to rigorous scientific evaluation (73). Interest in histamine has been heightened by the increasing evidence of key roles for mast cells in aberrant visceral pain associated with IBS (64,74). Food is one source of modulating histamine availability, but there are no studies to guide whether such strategies are beneficial. Food-associated salicylates are believed to be one of the more troublesome bioactive food chemicals (75), and a recent pilot cross-over study provided evidence in support (76).

### Functional foods

Functional foods are defined as foods that offer health benefits extending beyond basic nutrition. Whole foods or plant derivatives that have been evaluated in IBS and FC include antraquinones (senna, cascara, aloe, rhubarb) figs, kiwifruit, and prunes.

Anthraquinones are plant-based compounds derived from glycosides that are converted by bacterial glycosidases to poorly absorbed aglycones, which stimulate colonic motility and secretion (77,78). While the potential benefits of senna and cascara in patients with constipation are widely recognized, aloe and rhubarb are less well appreciated for their laxative properties. This is likely related to the paucity of data in patients with these conditions. A meta-analysis that included 3 RCTs and 151 patients with IBS of all subtypes reported a greater improvement in symptom score with Aloe vera vs placebo (standardized mean difference 0.41, *P* = 0.02 (79)). Another consecutive series of patients with IBS-C reported improvements in abdominal symptoms and stool frequency and consistency (80). There are no RCTs evaluating orally ingested rhubarb in patients with IBS or FC. An RCT from China in 374 patients with FC reported that a rhubarb plaster applied to the navel led to significant improvements in stool frequency and consistency (81).

Figs are a rich source of fiber and fructose, which can affect the colonic microbiota, production of short-chain fatty acids, stool consistency, and stool weight, all of which could influence bowel symptoms. A recent RCT from Iran compared rehydrated figs (90 g/d) or flixweed (60 g/d) with placebo for 4 months in 150 patients with IBS-C. Both interventions led to significant improvements in stool frequency, stool consistency, and the frequency but not severity of abdominal pain (82).

Kiwifruit come in green, gold, and red varieties and are rich in soluble (pectic polysaccharides) and insoluble (cellulose/hemicellulose) fibers, antioxidants, phytonutrients, and enzymes such as actinidin. Consequently, kiwifruit has been suggested to affect stool consistency, stool weight, colonic microbiota and short-chain fatty acids, mucosal immune function, and, perhaps, protein digestion (83). Numerous studies have found that 2 peeled kiwifruits per day can significantly improve stool frequency and stool consistency in patients with FC and IBS-C and reduce abdominal pain in patients with IBS-C (84–86).

Dried plums or prunes are a well-established natural laxative. The basis of such a laxative action may be its content of sorbitol, a sugar alcohol, which acts as an osmotic laxative, and/or its fiber content that includes pectin, cellulose, hemicellulose, and lignin. Dried apricots also contain sorbitol and fiber, though in smaller quantities than prunes (87). In RCTs, prunes in doses of 80–120 g/d (100 g = 12 prunes) significantly increase stool frequency and stool weight to a greater degree than placebo or psyllium (6 g/d) in patients with chronic constipation (88,89). In a 4-week, comparative effectiveness trial which enrolled 79 constipated patients from the United States, prunes (100 g/d), kiwifruit (2 fruits/d), and psyllium (12 g/d) led to significant increases in stool frequency compared with those in baseline. Although prunes led to the greatest increase in stool frequency, differences between the interventions were not statistically significant in this pilot study. Adverse events were most common with psyllium and least common with kiwifruit, perhaps because it is low in FODMAP content. At the end of treatment, a smaller percentage of participants were dissatisfied with kiwifruit compared with prunes or psyllium (*P* < 0.02) (86).

## CONCLUSION

In the past 10–15 years, diet has assumed an increasingly prominent role in our understanding and treatment of bowel disorders. The LFD has provided proof of concept for the effectiveness of diet interventions for patients with IBS. Despite its effectiveness, tolerability, acceptability, increased food costs, and nonresponse are all issues that create challenges for patients wanting to implement LFD. For these reasons, research to identify other effective diet interventions for bowel disorders are encouraged and eagerly awaited. As with almost all other aspects of bowel disorders, one size will not fit all patients. Just as restriction of FODMAPs is the beginning and not the end of the 3-phase LFD plan, we are at the beginning and not the end of the journey to find other evidence-based diet interventions for patients with bowel disorders. Further efforts to discover and validate biomarkers that identify patients who are more or less likely to respond to specific dietary interventions is another aspirational goal that will help us to step away from our current, highly imprecise, empiric treatment model and step toward the enticing concept of personalized nutrition.

## CONFLICTS OF INTEREST

**Guarantor of the article:** William D. Chey, MD.

**Specific author contributions:** All authors participated in the conception, preparation of the first draft, critical revision of subsequent drafts, and approval of the final manuscript.

**Financial support:** None to report.

**Potential competing interests:** P.S.: None. C.T. has received research funding from DSM Nutritional Products and Yakult Australia. P.R.G. has served as a consultant or advisory board member for Anatara, Atmo Biosciences, Falk Pharma, Immunic Therapeutics, Novozymes, Novoviah, Comvita, and Takeda. He has received research grants for investigator‐driven studies from Atmo Biosciences. He holds shares in Atmo Biosciences. His department financially benefits from the sales of a digital application, booklets, and online courses on the FODMAP diet. W.D.C. is a consultant for Abbvie, Allakos, Alnylam, Ardelyx, Arena, Bayer, Biomerica, Ironwood, Nestle, QOL Medical, Salix/Valeant, Takeda, Urovant Sciences, and Vibrant; has received grant and/or research study funding from Biomerica, Commonwealth Diagnostics International, QOL Medical, and Salix; has stock options in GI on Demand, Modify Health; serves on the Rome Board of Directors; and is a member of the Board of Trustees of the American College of Gastroenterology and Board of Directors of the International Foundation for Gastrointestinal Disorders.
